# TLR4: A Potential Therapeutic Target in the Experimental Pathogenesis of Hemolytic-Uremic Syndrome

**DOI:** 10.3390/ijms27187992

**Published:** 2026-09-08

**Authors:** Elisa Varrone, Veronica Pepe, Gianluca Storci, Massimiliano Bonafè, Barbara Brunetti, Maurizio Brigotti

**Affiliations:** 1 Department of Medical and Surgical Sciences (DIMEC), University of Bologna, 40126 Bologna, Italy; elisa.varrone2@unibo.it (E.V.); veronica.pepe7@unibo.it (V.P.); massimiliano.bonafe@unibo.it (M.B.); 2 IRCCS Azienda Ospedaliero-Universitaria di Bologna, 40138 Bologna, Italy; gianluca.storci@unibo.it; 3 Department of Veterinary Medical Sciences (DIMEVET), University of Bologna, Ozzano dell’Emilia, 40064 Bologna, Italy; b.brunetti@unibo.it

**Keywords:** hemolytic-uremic syndrome, Shiga toxins, Toll-like receptor 4, extracellular vesicles

## Abstract

Childhood infections by Shiga toxin (Stx)-producing *Escherichia coli* (STEC) cause bloody diarrhea and the life-threatening hemolytic-uremic syndrome (HUS). Stx2, produced by STEC in the gut, is released into the bloodstream and interacts with circulating cells (monocytes, neutrophils, and platelets) through two different receptors: Gb3Cer (globotriaosylceramide) and Toll-like receptor 4 (TLR4). Consequently, blood cells form leukocyte/platelet aggregates and release pathogenic Stx2-containing extracellular vesicles (EVs) that, by damaging the kidney, induce the transition from bloody diarrhea to HUS. Human blood was incubated with Stx2 to induce the formation of neutrophil/platelet and platelet-only aggregates (observed in May–Grünwald Giemsa-stained blood smears) and pathogenic blood cell–derived EVs (isolated by size-exclusion chromatography or differential centrifugation) toxic to Vero cells (assessed by a viability assay). In the presence of a monoclonal antibody against TLR4 (anti-TLR4), the formation of neutrophil/platelet and platelet-only aggregates was greatly reduced compared to treatment with Stx2 alone. Moreover, anti-TLR4 significantly decreased the total mass of pathogenic blood cell–derived EVs produced by Stx2 (detected by nanoparticle tracking analysis), which proved less toxic to Vero cells than those produced by Stx2 alone. The impairment of the Stx2-TLR4 axis obtained during the experimental pathogenesis of HUS provides proof-of-concept evidence supporting TLR4 as a potential therapeutic target.

## 1. Introduction

**Figure 1:**
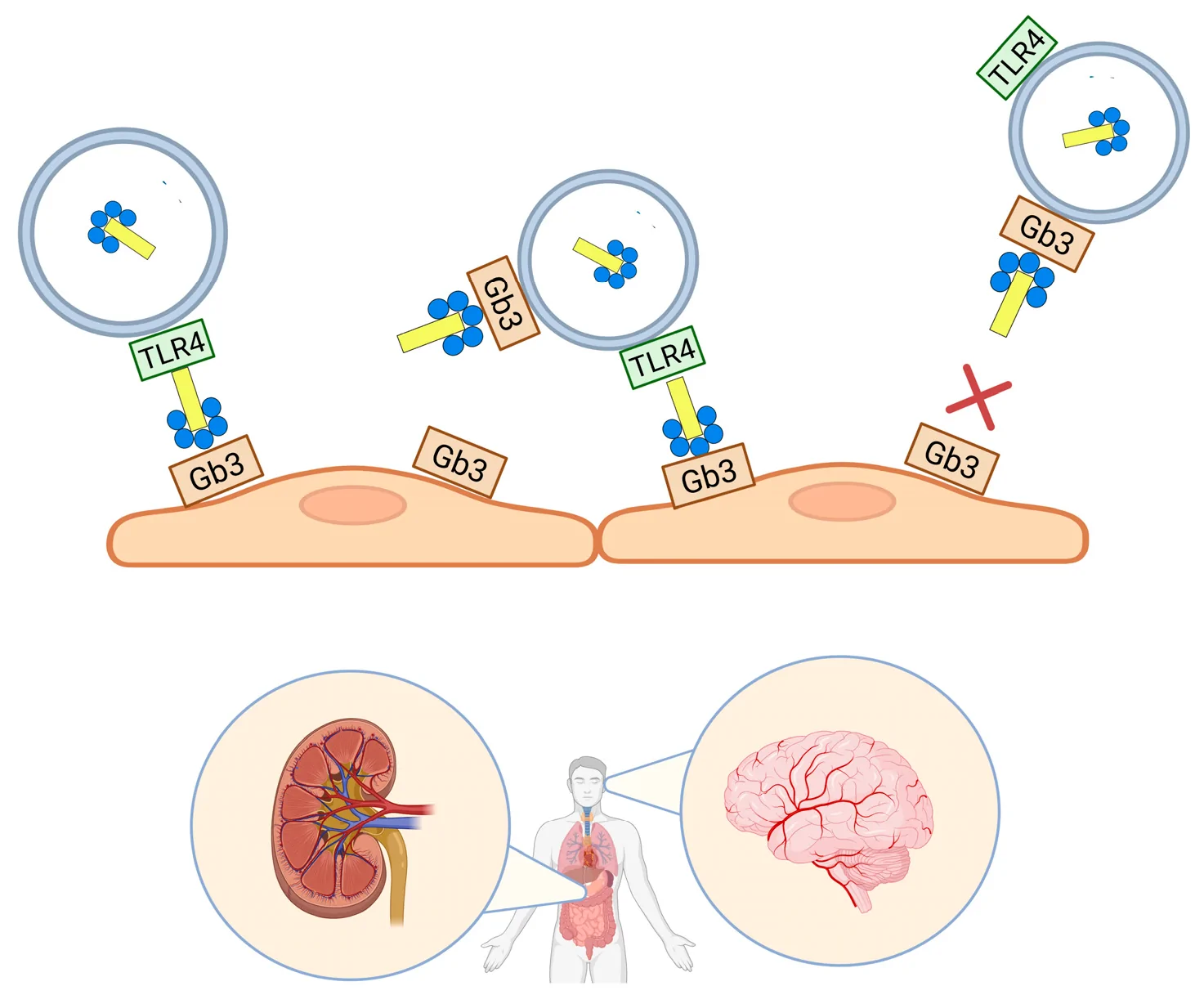
Role of TLR4-bound Stx2 with exposed B chains (blue) in the intoxication of target cells.

Hemolytic-uremic syndrome (HUS) is a rare and dangerous pathological condition that, in its typical form, is caused by infections with *Escherichia coli* strains producing Shiga toxins (Stxs), which are also called STEC (Stx-producing *E. coli*) [1,2,3,4]. The colonization of the gut by these bacteria is responsible for the intestinal phase of STEC infections, characterized by abdominal pain, vomiting, and diarrhea, often hemorrhagic. During the prodromal intestinal phase, toxins released into the bloodstream by the non-invasive STEC initiate a chain of events culminating in the HUS triad: thrombocytopenia, microangiopathic hemolytic anemia, and acute renal failure [1,2,3]. HUS is a thrombotic microangiopathy focused on the Stxs-induced damage of renal endothelial cells. The presence of a host of microthrombi within renal glomeruli decreases the number of platelets, reduces renal flow, and causes damage to passing erythrocytes, explaining the onset of the HUS triad in ~15% of young STEC-infected patients [1,2,3]. Indeed, HUS is considered the main cause of acute renal failure in children under the age of 3 years [5]. The simplest route for toxins to reach target endothelial cells would be to move in blood as a free molecule without interacting with blood cells or serum components. This is not the case since Stx2—the toxin type more frequently associated with HUS [5]—is neutralized by a protective blood factor (human serum amyloid P component; HuSAP) and is forced to find a different route for delivery [6,7,8]. Indeed, Stx2 binds to platelets, monocytes, and neutrophils by interacting with two receptors that differ in role and nature: the neutral glycolipid Gb3Cer (globotriaosylceramide) and the proteinaceous TLR4 (Toll-like receptor 4) [2,9]. Circulating cells are stimulated by these multiple interactions, and their response is twofold; i.e., the formation of circulating aggregates between platelets and leukocytes and the release of large extracellular vesicles (EVs) containing Stx2 and other virulence factors [10,11,12]. Although both structures have been found in the blood of patients during overt HUS [11,12], large pathogenic EVs were detected in the blood of STEC-infected children the day before the development of HUS [13]. Hence, EV-associated Stx2 is considered the most ominous form of the toxin that contributes to the pathogenesis of HUS. It is worth noting that Gb3Cer is not only expressed by circulating cells (platelets and monocytes) but also by target endothelial cells (and other renal cells), as it is responsible for their intoxication since it interacts with the five binding subunits of Stxs (B pentamer) [2]. On the other hand, TLR4 expressed by platelets, monocytes, and granulocytes (and derived EVs) could be considered an orienting receptor; i.e., since the toxic subunit of Stx2 (A chain) is responsible for the binding to TLR4 on the EV surface, the Gb3Cer-interacting B pentamer is exposed, thereby directing the pathogenic EVs toward target cells (Figure 1) [2,13]. Therefore, a given EV population should be considered harmful because of the presence of Stx2 (and other virulence factors), and in virtue of its ability to interact properly with target cells. In other words, EV toxicity depends on the cargo and on docking ability; i.e., two different EV populations with the same amount of associated virulence factors (e.g., Stx2) could affect the kidney differently based on the amount of TLR4-bound Stx2 on their surface [2,13]. In this light, TLR4 seems to be a good candidate for a focused therapeutic intervention aimed at reducing the extent of A chain–bound Stx2 on the EVs involved in HUS development.

TLR4 is a crucial element of the innate immune system that identifies invading pathogens by recognizing and binding to pathogen-associated molecular patterns such as bacterial lipopolysaccharide (LPS), thereby initiating an inflammatory response [14,15]. When LPS binds to LPS-binding protein (LBP) in human serum, the LPS-LBP complex can be recognized by CD14, which is expressed by monocytes/macrophages and neutrophils. Then, CD14-LPS binds to MD2, which stimulates the homodimerization of two TLR4 monomers and the subsequent recruitment and activation of the transcription factor nuclear factor-kB, and, as a consequence, the induction of pro-inflammatory mediators [16,17]. Platelets are TLR4-expressing cells that, in addition to their role in hemostasis, participate in innate immune and inflammatory responses. Upon adhesion, they change their shape and release molecules associated with their granules before the final step of platelet aggregation [18]. This process favors the release of bioactive mediators (CXCL4, CCL5, IL-1β, and serotonin) to trigger the immune response during hemostasis, and also fosters the physical interaction of platelets with neutrophils and leukocytes, mainly mediated by the interaction of platelet P-selectin with P-selectin granulocyte ligand-1 (PSGL1) as well as CD40 with the CD40 ligand (CD40L) [18]. Activation of platelets promotes the release of small and large EVs that have pro-coagulating activities and are involved in maintaining other physiological functions, such as stem cell homeostasis, healing, and immune surveillance. Platelet-derived EVs contain relevant biomolecules (DNA, RNA, lipids, proteins, and small metabolites) that play an important role in intercellular communication and in the pathogenesis of different human diseases [19]. TLR4 is involved in platelet activation and aggregation when stimulated by specific ligands, such as LPS [18].

Here, we show that targeting TLR4 in vitro during the experimental pathogenesis of HUS strongly reduced the formation of leukocyte/platelet and platelet-only aggregates, as well as the release of large pathogenic Stx2-containing EVs, hence protecting target Gb3Cer-expressing cells from intoxication.

## 2. Results

### 2.1. Targeting TLR4 with a Specific Antibody Impairs the Formation of Leukocyte/Platelet and Platelet-Only Aggregates by Stx2a

**Figure 2:**
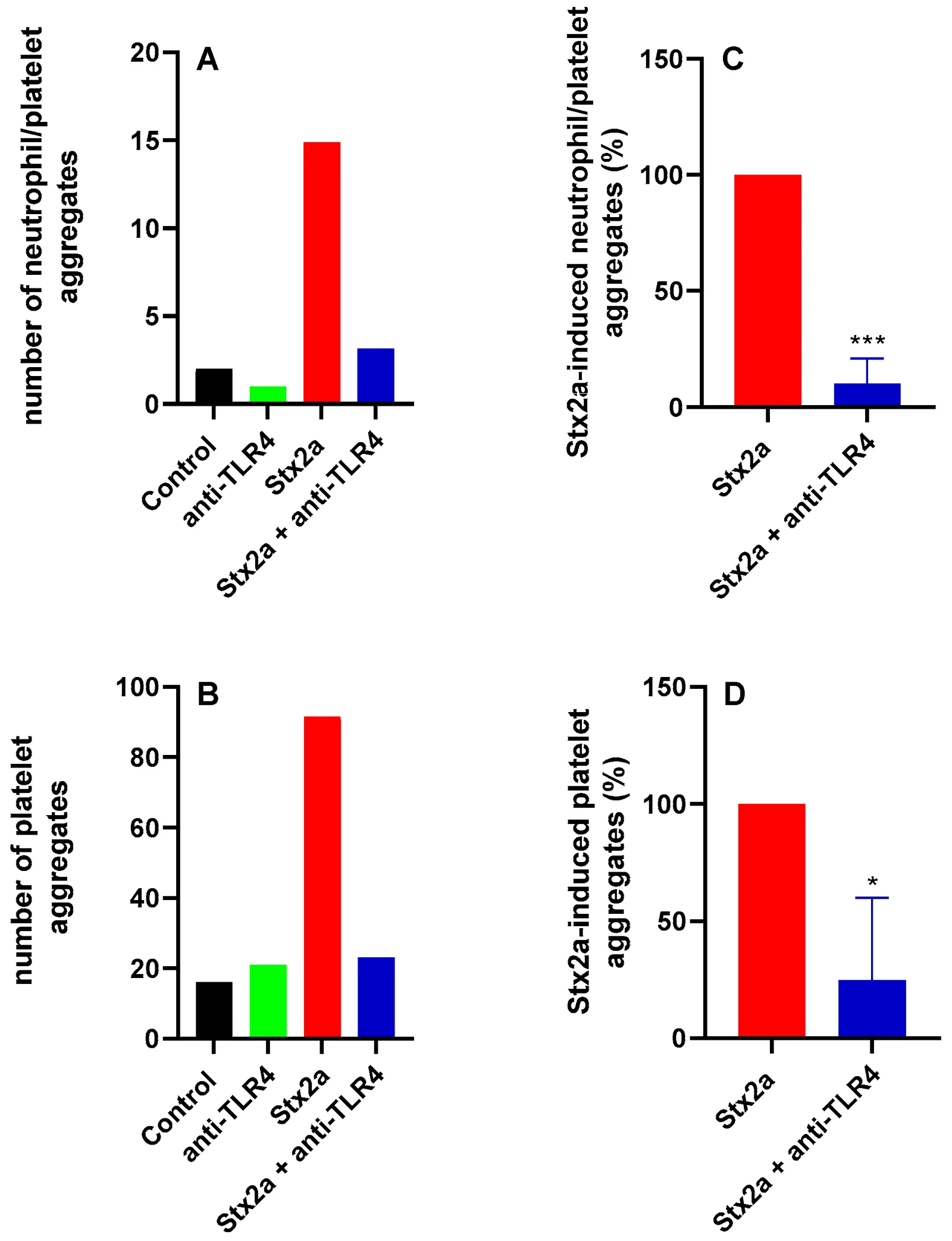
Effects of a monoclonal antibody to TLR4 on the formation of neutrophil/platelet or platelet-only aggregates induced by Stx2a. Blood samples from healthy donors were incubated for 4 h at 37 °C with Stx2a (2 nM) in the presence or absence of the mouse monoclonal anti-TLR4 antibody 3C3 (10 µg/mL). Then, the formation of neutrophil/platelet or platelet-only aggregates was assessed by optical microscopy on May–Grünwald Giemsa-stained blood smears. (**A**,**B**) Values obtained in a representative experiment performed with blood from a single donor are expressed as number of aggregates/100 polymorphonuclear leukocytes. (**C**,**D**) Values obtained with independent experiments with different human donors (*n* = 3) are expressed as percentage of total aggregates formed by Stx2a; data are means ± SD. * *p* < 0.05; *** *p* < 0.0001 (two-tailed unpaired *t*-test).

The formation of neutrophil/platelet aggregates (Figure S1A) or of clusters of platelets (Figure S1B) after treatment (4 h at 37 °C) of small aliquots of human blood with Stx2a (2 nM) was assessed by optical microscopy on May–Grünwald Giemsa-stained blood smears. In the representative experiment shown in Figure 2A,B, the number of neutrophil/platelet or platelet-only aggregates per 100 polymorphonuclear leukocytes was assessed in the blood from a single donor. The addition of the anti-TLR4 monoclonal antibody 3C3 during the toxin challenge induced a dramatic inhibitory effect on aggregate formation, while the controls were poorly affected. The strong antibody-dependent reduction in the number of Stx2a-triggered aggregates (set at 100%) was consistently observed in independent experiments with different donors (*n* = 3), resulting in a significant decrease in the percentage of aggregates induced by toxin treatment (Figure 2C,D).

### 2.2. Targeting TLR4 with a Specific Antibody Impairs the Formation of Blood Cell–Derived EVs by Stx2a

**Figure 3:**
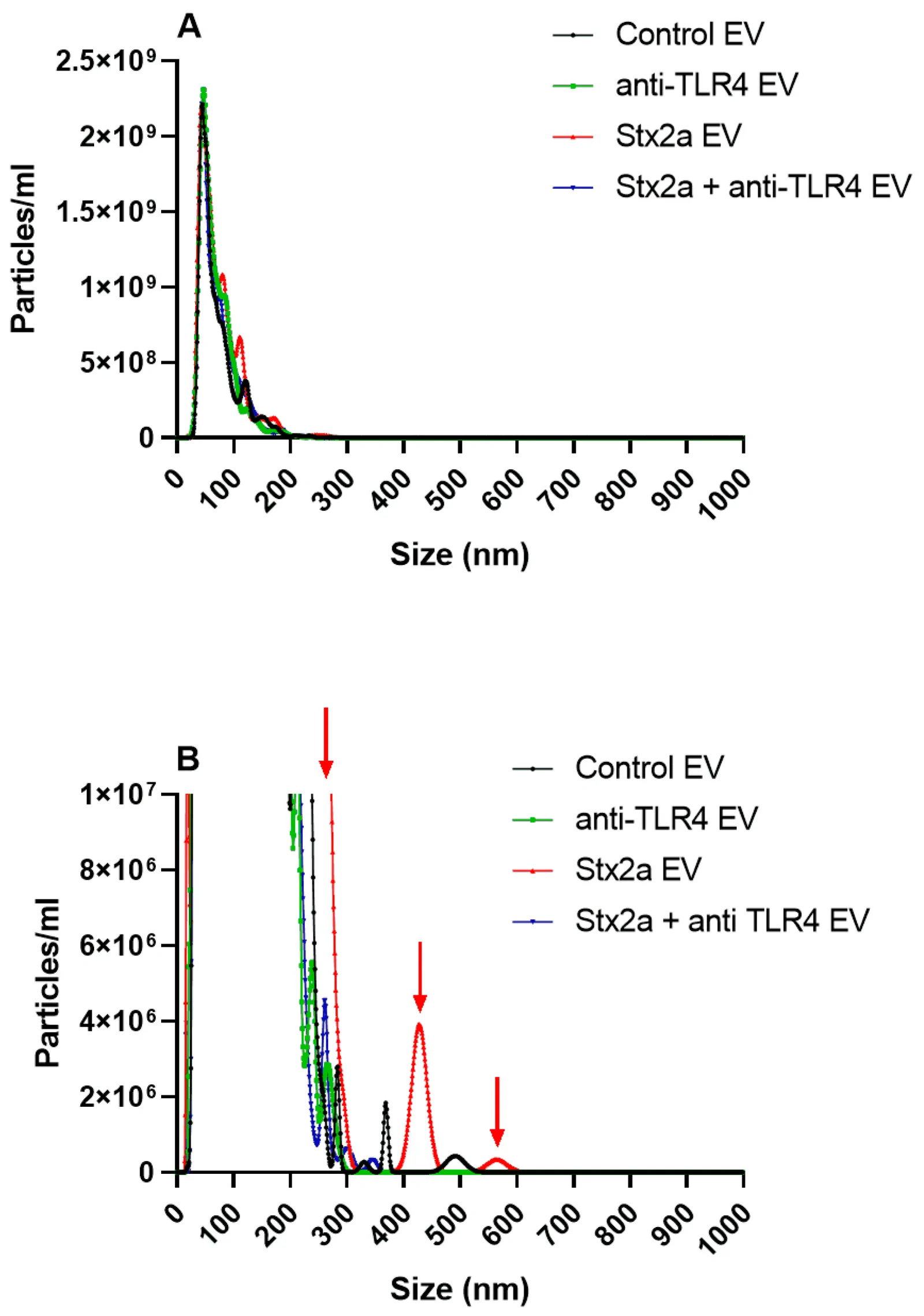
Determination of the size and number of EVs by nanoparticle tracking analysis. EVs were obtained after treatment of human blood from a single representative donor with Stx2a (2 nM) in the absence (red line) or presence (blue line) of the mouse monoclonal anti-TLR4 antibody 3C3 (10 µg/mL), by treatment with vehicle (black line), or with the antibody alone (green line). The vesicles were isolated by gel filtration size-exclusion chromatography as described in the Materials and Methods Section—100-fold diluted and applied to NanoSight. (**A**,**B)** Data are expressed as number of particles/mL for the different EV populations, differing from each other in diameter by 0.5 nm. Red arrows indicate the newly emerging large-vesicle components. The same data are reported in panels (**A**,**B**), which differ only in the scale of the y-axis.

To investigate the most dangerous phase of HUS pathogenesis, when Stx2a induces the release of large pathogenic EVs by circulating cells, whole blood from a single representative donor (750 µL) was challenged with Stx2a in the presence or absence of the anti-TLR4 monoclonal antibody, as described above. At the end of the incubation, blood cells and apoptotic bodies were removed by centrifugation, and the sample was applied to qEV original 70 nm columns (size-exclusion chromatography), collecting 500 µL fractions. According to the column performance described by the manufacturer and based on the elution profiles previously obtained in similar experiments [20], fractions 6–9—which contain the blood cell–derived EVs—were merged. The analysis of the Brownian motions of the EVs present in the preparations (nanoparticle tracking analysis) allows for the determination of the size and number of the different vesicle populations, revealing the presence of new discrete peaks induced by the treatment with Stx2a, featuring at 275 nm, 425 nm, and 550 nm (Figure 3A,B, red line vs. black line). These new vesicle populations were not observed when blood samples were treated with the toxin in the presence of the anti-TLR4 antibody (Figure 3A,B, red line vs. blue line).

**Figure 4:**
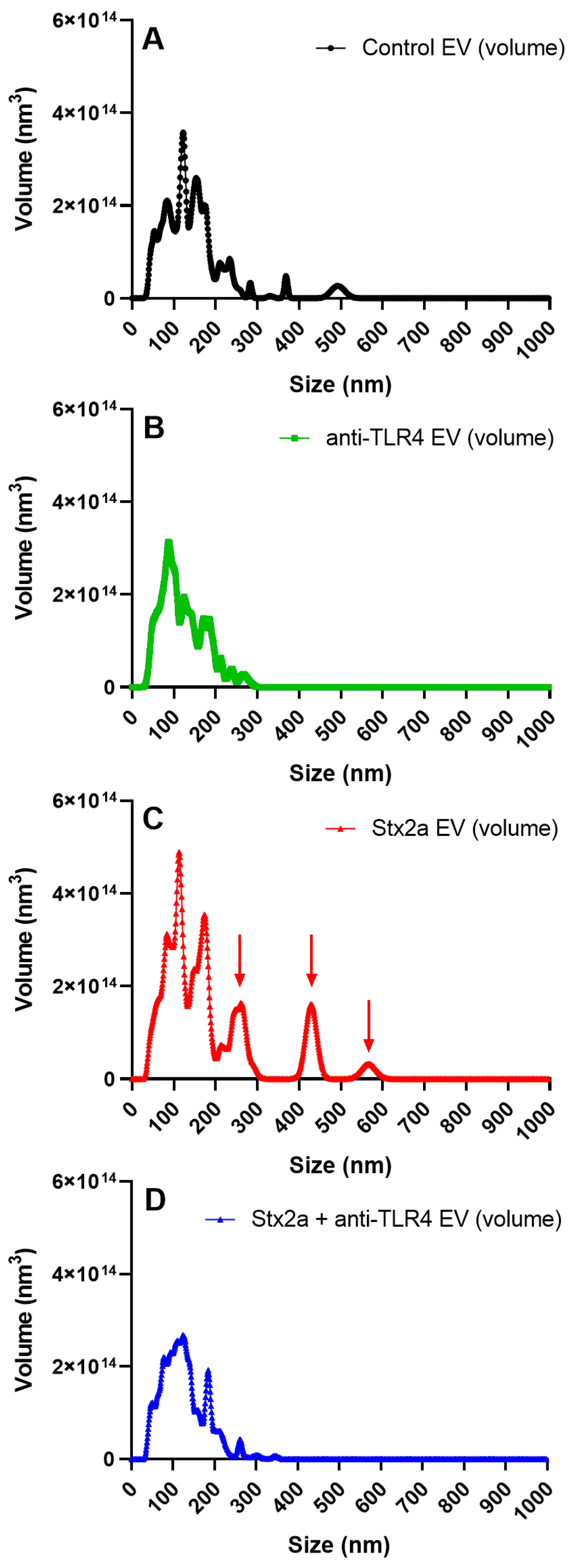
Determination of EV volume by nanoparticle tracking analysis. EVs were obtained after treatment of human blood from a single representative donor with Stx2a (2 nM) in the absence ((**C**), red line) or presence ((**D**), blue line) of the mouse monoclonal anti-TLR4 antibody 3C3 (10 µg/mL), by treatment with vehicle ((**A**), black line), or with the antibody alone ((**B**), green line). The vesicles were isolated by gel filtration size-exclusion chromatography as described in the Materials and Methods Section—100-fold diluted and applied to NanoSight. The data reported in Figure 3 (number of particles/mL) were used to calculate the total volume (nm^3^) of each population of EV according to their diameter, assuming a spherical shape. Red arrows indicate the newly emerging large-vesicle components.

**Figure 5:**
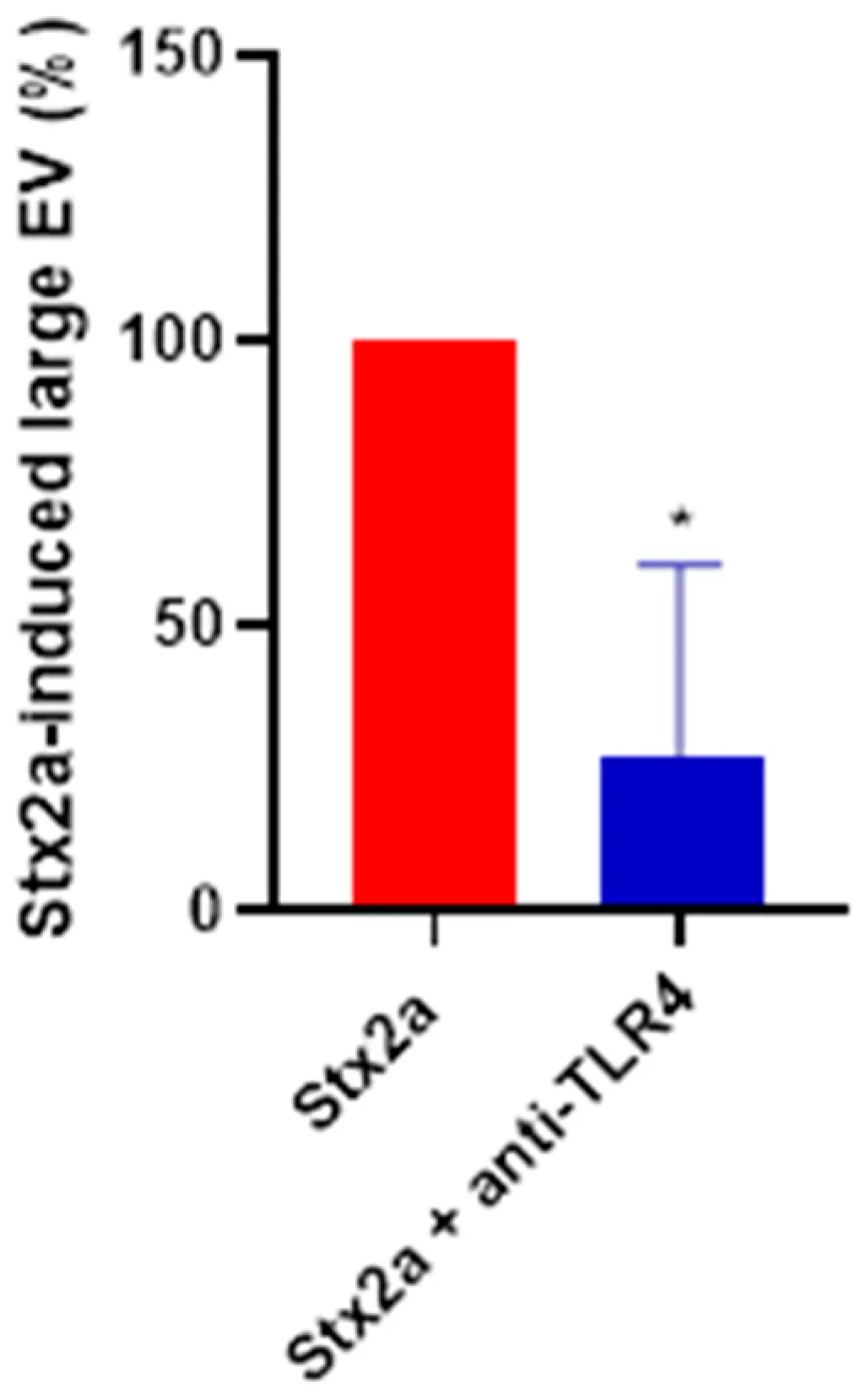
Significant inhibition of the production of large (>200 nm) Stx2a-triggered EVs by a monoclonal antibody to TLR4. EV isolation and the determination of EV volume by nanoparticle tracking analysis are as in Figure 4. Data obtained from independent experiments with blood from different human donors (*n* = 3) are expressed as percentage (mean ± SD) of the stimulation obtained in the presence of Stx2a, which was set at 100%. Data are means ± SD. * *p* < 0.05 (two-tailed unpaired *t*-test).

If the volume of the different vesicle populations is calculated instead of considering only their number (Figure 4), the larger EV populations (>200 nm diameter) account for 16% of the EVs found in the Stx2a-stimulated samples (Figure 4C, red arrows)—in keeping with the notion that large-sized EVs are found in the blood of STEC-infected patients developing HUS [11,13]. In the presence of the anti-TLR4 antibody (Figure 3 and Figure 4, blue lines), the number and the volume of the >200-nanometer-diameter EVs released by the toxin were strongly reduced compared with those obtained by Stx2a alone. Moreover, the total volume of EVs obtained in the presence of Stx2a (5.45 × 10^16^ nm^3^) is higher than that measured in controls (3.39 × 10^16^ or 2.94 × 10^16^ nm^3^, without or with the anti-TLR4 antibody, respectively) and clearly dropped to the control level in the presence of the combined treatment, Stx2a/anti-TLR4 antibody (3.01 × 10^16^ nm^3^). These findings were consistently observed in independent experiments performed with different analyzed donors (*n* = 3), showing a significant impairment of Stx2a-dependent >200-nanometer EV release when TLR4 is targeted by the specific antibody (Figure 5).

### 2.3. EVs Obtained by Human Blood Challenged with Stx2a in the Presence of a Specific Antibody to TLR4 Are Less Toxic to Eukaryotic Cells

**Figure 6:**
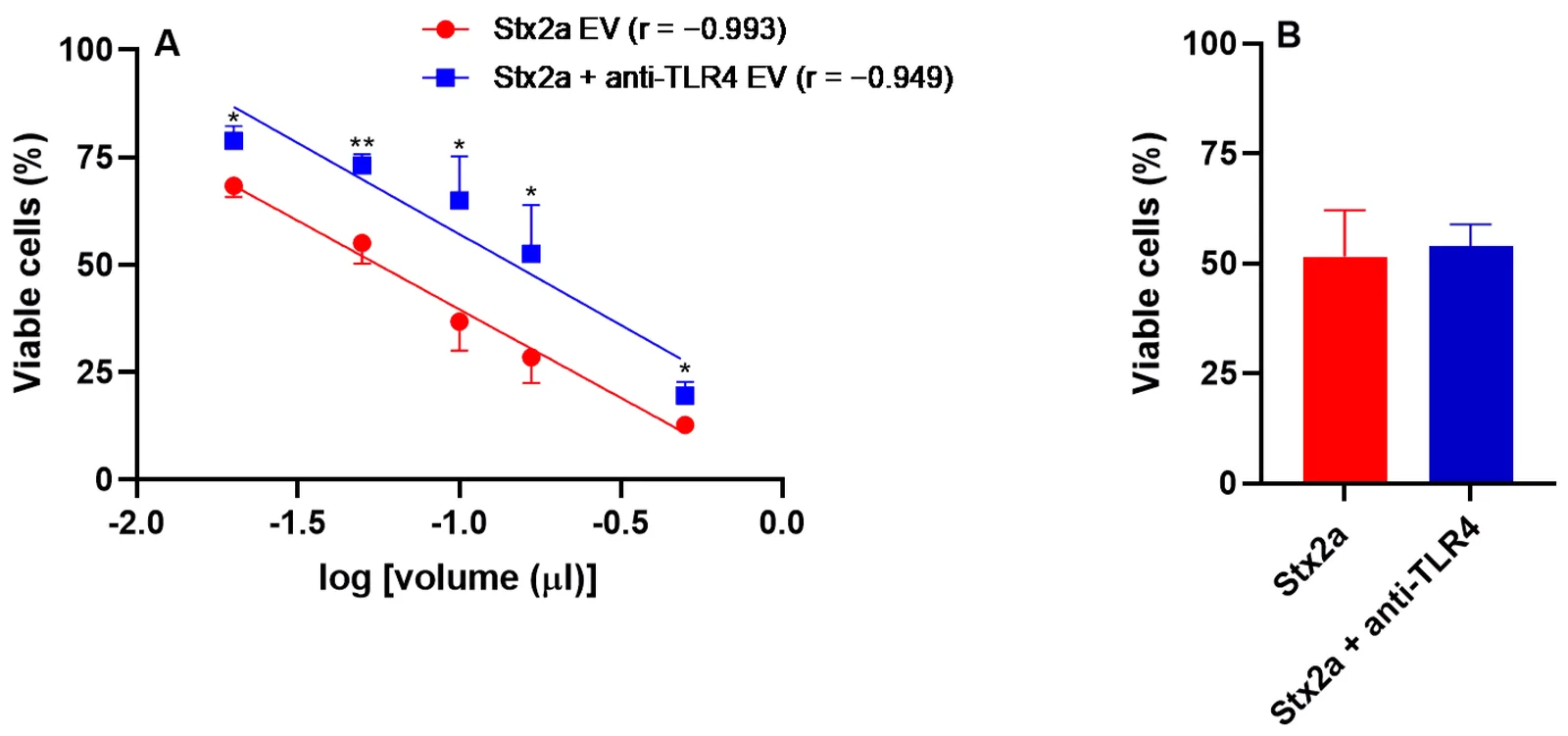
Reduced cytotoxicity of EVs obtained by human blood challenged with Stx2a in the presence of a specific antibody to TLR4: (**A**) EVs were obtained after treatment of human blood from a single representative donor with Stx2a (2 nM) in the absence (red line) or presence (blue line) of the mouse monoclonal anti-TLR4 antibody 3C3 (10 µg/mL). The vesicles were isolated by differential centrifugation, as described in the Materials and Methods Section, and different aliquots of the preparations were added to cultured Gb3Cer-expressing Vero cells. The toxic effects were measured by the viability assay described in the Materials and Methods Section. Data are expressed as the mean percentage of viable cells ± SD obtained in three independent experiments, performed in triplicate, with respect to controls carried out without vesicles; the reported straight lines significantly differed in the y-intercept (*p* < 0.005); Pearson’s correlation coefficient (r) was used to assess the correlation between variables. Control EVs derived from human blood treated with PBS or with the antibody alone did not elicit any toxic effects on Vero cells. * *p* < 0.05; ** *p* < 0.01 (two-tailed unpaired *t*-test). (**B**) Vero cells were treated with free Stx2a (0.15 pM) with (blue column) or without (red column) the mouse monoclonal anti-TLR4 antibody 3C3 (21 µg/mL) in an experiment performed in triplicate. Data are expressed as mean percentage of viable cells ± SD (*n* = 3) with respect to controls carried out with PBS-BSA 1%.

To obtain amounts of pathogenic EVs sufficient to intoxicate Stx-sensitive cells, greater volumes of human blood (10 mL) obtained from a single donor were treated as described above, and the large blood cell–derived EVs, which sedimented between 10,400× and 20,800× *g*, were isolated by centrifugation. Aliquots of the different EV preparations were added to Gb3Cer-expressing Vero cells, and their effects were assessed by a viability assay after 72 h of incubation. Large EVs released by human circulating cells after the combined treatment with Stx2a/anti-TLR4 antibody were ~3-fold less toxic than those produced by blood cells treated with Stx2a alone (IC_50_ 0.148 µL vs. IC_50_ 0.056 µL) (Figure 6A), despite the lack of effect of the same antibody on free Stx2a intoxicating Vero cells (Figure 6B). We conclude that targeting TLR4 during a crucial phase of HUS pathogenesis—such as the stimulation of blood cells by Stx2a—reduced the number, mass, and toxic cargo of the EVs produced and released by these cells, clearly demonstrating a protective effect in ex vivo settings.

## 3. Discussion

The main virulence factors responsible for the development of HUS in STEC-infected patients are Stxs that target only a restricted subset of cells endowed with the receptor Gb3Cer; i.e., endothelial cells of the microvasculature of the kidney, brain, and intestine; epithelial cells of renal glomeruli; and tubular epithelial cells and mesangial cells of the kidney [1,2,9,10]. The focused distribution of Gb3Cer explains the specific targeting of the gut during the prodromal intestinal phase of STEC infections and of the kidney during HUS [21]. This is a key point highlighting the relevance of this receptor in the pathogenesis of HUS.

Given the pivotal role of Gb3Cer as a targeting receptor, it should also be considered that the journey of Stxs from the intestinal lumen to the kidney is not direct and linear. Two factors complicate the delivery of Stx2 to the kidney: the binding of the toxin to the soluble neutralizing molecule HuSAP, and the interactions of the toxin with platelets, neutrophils, and monocytes involving not only Gb3Cer but also TLR4 [2]. These multiple interactions induce the formation of blood cell aggregates formed by circulating cells. The consequent release of blood cell–derived pathogenic EVs containing Stx2 is followed by their docking to the Gb3Cer-expressing target cells, which seems to be dependent on the role of TLR4 as an orienting receptor. As shown in Figure 1, TLR4, by interacting with the A chain of Stx2 on the EV surface, promotes the exposure of the Gb3Cer-interacting B chains of the toxin, allowing for intoxication.

In the present paper, we have demonstrated that targeting TLR4 with a specific monoclonal antibody reduced the formation of neutrophil/platelet and platelet-only aggregates and, more importantly, of the Stx2-containing EVs that are considered the most harmful form of the toxin during the pathogenesis of HUS. Gb3Cer-expressing Vero cells are protected when EVs are derived from human blood treated with Stx2 in the presence of the anti-TLR4 antibody. Although it is surprising that impairing TLR4 action without blocking the main Gb3Cer receptor would induce protective effects on cells targeted by the pathogenic EVs, these findings are in line with previous investigations. In collaboration with Northern Antibiotic (Helsinki, Finland), we have developed a drug aimed at interacting with Stx2 in patients’ blood to prevent binding to TLR4. The therapeutic molecule, named NAB815, is the non-toxic form of the old antibiotic polymyxin B [22] and is effective in blocking the interaction of Stx2 with TLR4 but not with Gb3Cer [23]. NAB815 proved to be effective in reducing the amount and toxic content of EVs derived from human blood treated with Stx2. These EVs were less toxic in cellular and animal models compared with EVs produced by Stx2 alone [23]. Strikingly, the drug also protected mice intoxicated with free Stx2, showing reduced mortality, decreased weight loss, and milder histopathological changes associated with better renal function compared to mice treated with the toxin alone [23]. The effect is directly related to the specific binding of the drug to Stx2 and highlights the relevance of endogenous pathogenic EVs produced by murine circulating cells during Stx2 challenge. In other words, targeting the receptor (TLR4) by a specific antibody or the ligand (Stx2a) by a specific drug induced similar protective effects.

In this context, it is important to note that LPS—the main ligand of TLR4—plays an important role in the development of HUS since synergy between LPS and Stx2 has been shown to exacerbate renal toxicity [14]. In our experimental setting, the only source of LPS is the very low amount of endotoxin (7.4 ng/mg of protein) contaminating our Stx2 preparation. Additionally, in our experimental setting, Stx2 (2 nM) is roughly 150 ng/mL, corresponding to about 1 pg/mL LPS. However, human blood stimulated with 1 pg/mL LPS showed no formation of leukocyte/platelet complexes [24], and immune cells stimulated with 30 pg/mL (neutrophils) or 100 pg/mL (monocytes) LPS showed no significant release of the chemokine CXCL8 with respect to controls [24,25]. Given this scenario, the engagement of TLR4 by LPS under our experimental conditions is unlikely.

Since interfering with the Stx2-TLR4 axis during the experimental pathogenesis of HUS seems promising, and given that TLR4 has been exploited as a pharmacological target, other potential drugs could be considered. Several TLR4 antagonists and inhibitors have been produced in the past years [16,26,27] and studied in animal models, even though they have not been licensed for clinical use. For example, TLR4 antagonists have demonstrated efficacy in models of influenza, sepsis, vascular inflammation, diabetic nephropathy, and inflammatory bowel disease; however, they did not prove to be effective in clinical trials [16,28]. NI-0101 is a humanized monoclonal antibody that binds TLR4, preventing signal transduction; however, it did not demonstrate protective effects in a Phase II rheumatoid arthritis trial [29]. MTS510 and Sa15-21 are two mouse antibodies that target the TLR4-MD2 complex rather than TLR4 [26]. CRX-526 is a synthetic TLR4 antagonist whose administration has been shown to prevent renal injury in diabetic nephropathy and the development of severe inflammatory bowel disease in murine models [16,26]. Eritoran is a synthetic lipid A analog that, by binding to the TLR4-MD2 complex, dampens excessive inflammatory responses observed in animal models of liver fibrosis, cardiac hypertrophy, myocardial ischemia-reperfusion damage, trauma, and sepsis. Nevertheless, human clinical trials with this compound were not successful [16,28]. Similarly, the small-molecule inhibitor TAK-242, which strongly binds to TLR4, has shown promising results in disease prevention in animals but has not demonstrated efficacy in humans [16,28]. A group of monosaccharide-based antagonists of TLR4 was named FP. One of them is FP7, a di-phosphorylated monosaccharide that was found to reduce inflammation in vascular tissue and to enhance survival in animal models of sepsis and influenza; still, it has not yet reached the human clinical trial stage [16,28]. Similarly, FP12 has also not yet advanced to clinical trials, although it has been demonstrated to reduce lung inflammation in mouse models of influenza [16].

Despite the variety of compounds targeting TLR4, it cannot be taken for granted that a specific TLR4 antagonist would also prevent the binding of Stx2 to the same receptor—a prerequisite for use in STEC-infected patients. Carefully conducted studies recapitulating in vitro the crucial step of the pathogenesis of HUS, such as the release of pathogenic EVs after toxin challenge of human blood, will reveal the best compound to be considered. During the early stages of STEC-induced HUS, activation of a strong inflammatory response culminating in the release of cytokines and chemokines by innate immune cells and activation of complement and thrombotic cascades have been demonstrated [14]. Administration of this class of compounds could also be beneficial in reducing the inflammatory burden of patients, besides the direct effect on toxin action. On the other hand, even if TLR4 antagonists prove ineffective in controlling the inflammatory response in STEC-infected patients, they could be beneficial in preventing more direct toxin actions, such as the release of pathogenic EVs. However, it is worth noting that the present study is mainly preclinical since it is based on an ex vivo human blood model. Further studies using disease-relevant renal or animal models would be useful before clinical translation is considered. Finally, it has not escaped our notice that the findings described in the present paper further strengthen the relevance of NAB815 as a possible tool in the prevention of HUS, considering the ineffectiveness of the studied TLR4 antagonists in human clinical trials.

## 4. Materials and Methods

### 4.1. Toxin

Stx2a was purified from *E. coli* C600-933W (a generous gift by Dr. Alison O’Brien; Department of Microbiology and Immunology, Uniformed Services University of the Health Sciences, Bethesda, MD, USA). Stx2a was isolated through affinity chromatography (1.5 × 0.6 cm column, 1 mL in volume) with activated Sepharose coupled to bovine serum albumin (BSA) linked to a terminal galabiose (Galα1-4Galβ-O-spacer—BSA, Glycorex, Lund, Sweden) as an analog receptor [30]. Then, the isolated toxin was applied to an ActiClean Etox column (Sterogene Bioseparations, Carlsbad, CA, USA) to reduce LPS contamination. The purified Stx2a was quantified by the Lowry assay and tested using the ToxinSensorTM Gel Clot Endotoxin Assay Kit (GenScript, Piscataway, NJ, USA), showing a low amount of contaminating LPS (7.4 ng/mg Stx2a). Stx2a was kept in aliquots at −80 °C. To prevent nonspecific interactions with plastic tubes, the toxin was diluted with phosphate-buffered saline (PBS) containing 1% BSA.

### 4.2. Human Blood Collection

After obtaining written informed consent, human blood anticoagulated with a citrate–phosphate–dextrose solution was obtained from three healthy adult donors (Emilia-Romagna Regional Blood Centre, Maggiore Hospital, AUSL, Bologna, Italy). The median age of the donors was 40.0 years (range: 25–63), and the AB0/Rh groups were A−, 0+, and 0−. Blood samples (300 µL) were used for the experiments on the determination of leukocyte/platelet and platelet-only aggregates in blood smears. In contrast, larger blood samples (750 µL or 10 mL) were collected for the isolation of blood cell–derived extracellular vesicles.

### 4.3. Formation and Detection of Neutrophil/Platelet and Platelet-Only Aggregates

Human blood samples from three different donors (300 µL) were treated with 2 nM Stx2a in the presence or absence of 10 µg/mL of the mouse monoclonal anti-TLR4 antibody (HM2247 3C3, Hycult-Biotech, Uden, The Netherlands) or vehicle for 4 h at 37 °C to allow the formation of leukocyte/platelet or platelet-only aggregates. Then, 1.5 µL of blood was smeared, stained with May–Grünwald Giemsa, and neutrophil/platelet and platelet-only aggregates/100 polymorphonuclear leukocytes were counted using an optical microscope at 100× magnification.

### 4.4. Isolation of Blood Cell–Derived Extracellular Vesicles

Human blood cell–derived extracellular vesicles were isolated by gel filtration size-exclusion chromatography with qEV original 70 nm columns (Izon, Christchurch, New Zealand), according to the manufacturer’s instructions. Blood samples (750 µL) from three different donors were diluted with an equal volume of Dulbecco’s Modified Eagle Medium (DMEM, high glucose, w/o L-glutamine, and with sodium pyruvate; Capricorn Scientific, Ebsdorfergrund, Germany) supplemented with Gly–Pro–Arg–Pro (GPRP, 10 µM; Merck, Darmstadt, Germany) as a fibrin polymerization inhibitor, and incubated for 4 h at 37 °C with 2 nM Stx2a in the presence or absence of the anti-TLR4 monoclonal antibody described above (10 µg/mL) or vehicle. Then, samples were centrifuged at 2600× *g* for 35 min to discard blood cells and platelets, followed by centrifugation at 10,400× *g* for 25 min to remove apoptotic bodies. The supernatant (500 µL) was applied to the above-mentioned columns equilibrated with PBS, and then 20 fractions (500 µL each) were collected by eluting the column with the same buffer. Fractions 6 to 9 were pooled and analyzed by nanoparticle tracking analysis.

For cytotoxicity experiments, EVs were obtained by incubating very large human blood samples (10 mL) diluted with an equal volume of DMEM and incubated as described above. EVs were isolated by differential centrifugation: the first centrifugation took place at 2600× *g* for 35 min, as previously described; the collected plasma was diluted with an equal volume of DMEM and centrifuged at 10,400× *g* for 25 min; finally, the EV-containing supernatant was centrifuged at 20,800× *g* for 1 h to obtain large EVs (10,400–20,800× *g*). EVs were washed with 500 µL of PBS, centrifuged at 20,800× *g* for 40 min, and the pellets were then resuspended in 17 µL of PBS and frozen at −80 °C for the viability assays.

### 4.5. Nanoparticle Tracking Analysis

Nanoparticle tracking analysis (NTA) was performed using the NanoSight RS300 system (Malvern Panalytical, Malvern, UK) to quantify the size and relative abundance of the isolated EVs. Each sample was diluted 1:100 with 0.1-micrometer-filtered PBS, and the average number of particles and concentration (particles/mL) were determined by recording and analyzing three 60 s movies. NTA 3.4 analytical software was used to characterize each sample. Instrument characteristics and settings: camera, NSCMOS; laser, 532 nm; syringe pump, flow rate 30 (a.i.); screen gain, 1; camera level, 15–16; detection threshold, 3–4; particles/frame during acquisition, above 18–20 particles/frame.

### 4.6. Vero Cell Viability Assay

Gb3Cer-expressing Vero cells were kindly provided by Dr Stefano Morabito (European Reference Laboratory for *Escherichia coli*, Istituto Superiore di Sanità, Rome, Italy) and cultured in DMEM supplemented with 10% FBS, 2 mM L-glutamine, and 1% penicillin–streptomycin at 37 °C in a water-saturated 5% CO_2_ atmosphere in air. To test the toxic effect of the large blood cell–derived EVs isolated by differential centrifugation, cells (5 × 10^3^) were resuspended in 100 µL of medium, seeded in 96-well plates, and incubated for 24 h. Then, cells were treated with different volumes (0.02–0.5 µL) of the isolated EVs and, after 72 h, 100 µL of Cell Titer-Glo Reagent was added to each well to measure the percentage of live cells by a luminescent cell viability assay (Cell titer-Glo, Promega, Milan, Italy), according to the manufacturer’s instructions. The assay specifically quantifies the amount of ATP as a product of live cells.

### 4.7. Statistical Analysis

GraphPad Prism version 8 was used for statistical analysis. After determining whether the distribution of continuous data (means and SD) was normal, differences were examined using the unpaired *t*-test. Correlations between variables were assessed by Pearson’s correlation coefficient. A *p*-value < 0.05 was considered statistically significant.

## Data Availability

The data presented in this study are available upon request from the corresponding author.

## Supplementary Materials

The following supporting information can be downloaded at: https://www.mdpi.com/article/10.3390/ijms27187992/s1.

## Abbreviations

The following abbreviations are used in this manuscript:

HUS	Hemolytic-uremic syndrome
Stx	Shiga toxin
Stx2	Shiga toxin 2
STEC	Stx-producing *Escherichia coli*
HuSAP	Human serum amyloid P component
Gb3Cer	Globotriaosylceramide
TLR4	Toll-like receptor 4
EV	Extracellular vesicle
LPS	Bacterial lipopolysaccharide
LBP	LPS-binding protein
PSGL1	P-selectin granulocyte ligand-1
CD40L	CD40 ligand
BSA	Bovine serum albumin
PBS	Phosphate-buffered saline
