# Supplementary material for: TLR4: A Potential Therapeutic Target in the Experimental Pathogenesis of Hemolytic-Uremic Syndrome
Source: Int J Mol Sci. 2026 Sep 8;27(18):7992. doi: 10.3390/ijms27187992 (PMC13607936; doi:10.3390/ijms27187992)
Supplement: Supplementary file 1 [file ijms-27-07992-s001.zip › ijms-4520497-supplementary.pdf]

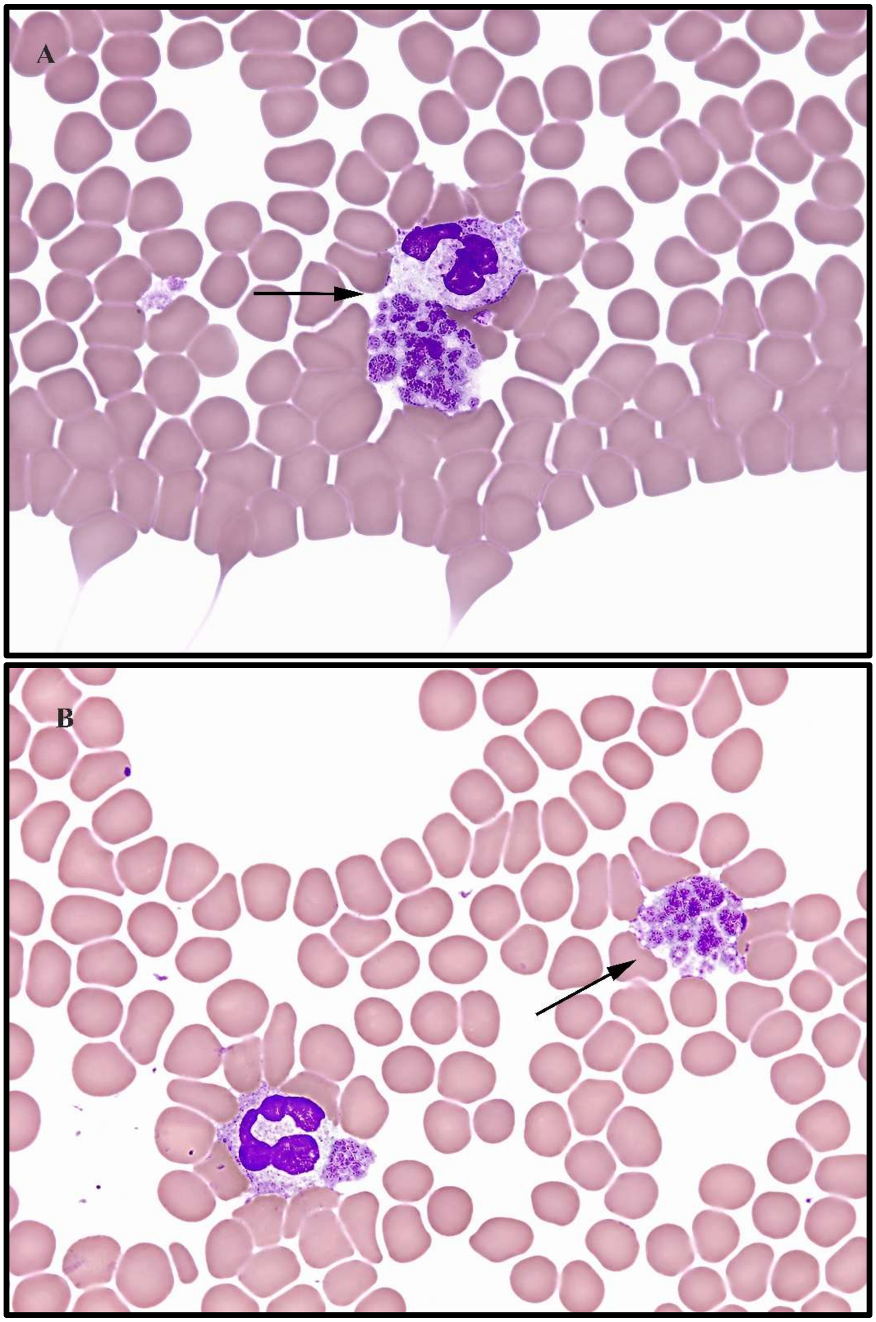

Figure S1. Peripheral blood smears after treatment with Shiga toxin 2. Blood samples from a representative donor were treated with 2 nM Stx2 as described in Materials and Methods. The micrographs show a neutrophil adhering to platelets (**A**) and a cluster of platelets (**B**) (May-Grünwald Giemsa stain, 100x magnification).
